# High-risk HPV infection and the risk of preterm birth in a Romanian tertiary maternity cohort: a prospective observational study

**DOI:** 10.25122/jml-2025-0102

**Published:** 2025-07

**Authors:** Carmen Elena Condrat, Dragos Cretoiu, Simona Raluca Iacoban, Silviu Cristian Voinea, Nicolae Suciu

**Affiliations:** 1 Department of Obstetrics and Gynecology, Carol Davila University of Medicine and Pharmacy, Bucharest, Romania; 2 Department of Obstetrics and Gynecology, Alessandrescu-Rusescu National Institute for Mother and Child Health, Bucharest, Romania; 3 Department of Medical Genetics, Carol Davila University of Medicine and Pharmacy, Bucharest, Romania; 4 Materno-Fetal Medicine Excellence Center, Alessandrescu-Rusescu National Institute for Mother and Child Health, Bucharest, Romania; 5 Department of Obstetrics and Gynecology, Suceava County Hospital, Suceava, Romania; 6 Department of Oncological Surgery, Carol Davila University of Medicine and Pharmacy, Bucharest, Romania; 7 Department of Oncological Surgery, Alexandru Trestioreanu Oncology Institute, Bucharest, Romania

**Keywords:** premature birth, papillomavirus infections, pregnancy complications, infectious, pregnancy, cohort studies, prospective studies, risk factors, Romania

## Abstract

Preterm birth (PTB; < 37 weeks) affects ^2^10 % of pregnancies and is the leading cause of neonatal mortality. Whether maternal high-risk human papillomavirus (hr-HPV) infection contributes to spontaneous PTB is unsettled. Romania, with Europe’s highest cervical-cancer burden, offers a relevant setting to explore this association. We prospectively followed 151 women enrolled before 14 weeks’ gestation at a tertiary maternity hospital (January 2021–May 2022). Cervical samples were tested with the Cepheid Xpert HPV assay, which detects 14 high-risk HPV types (16, 18, 31, 33, 35, 39, 45, 51, 52, 56, 58, 59, 66, 68). Maternal age, parity, smoking, body mass index, and comorbidities were recorded. PTB was defined as delivery < 37 weeks (very PTB < 34 weeks). Multivariable logistic regression estimated adjusted odds ratios (aOR) for PTB associated with hr-HPV, and Kaplan–Meier curves compared time-to-delivery between infected and uninfected pregnancies. hr-HPV DNA was detected in 60/151 pregnancies (39.7 %). PTB occurred in 28.3 % of hr-HPV-positive versus 13.2 % of hr-HPV-negative women (*P* = 0.02); very PTB rates were 8.3 % and 2.2 %, respectively. Median gestational age and birth weight were lower among infected mothers (38.0 weeks vs 39.0 weeks, *P* = 0.04; 3025 g vs 3230 g, *P* = 0.03), while Apgar scores were comparable. After adjustment for maternal covariates, hr-HPV remained independently associated with PTB (aOR = 2.38; 95% CI 1.07–5.29; *P* = 0.033), and survival analysis confirmed a higher cumulative hazard of early delivery (log-rank *P* = 0.021). First-trimester hr-HPV carriage approximately doubled the odds of preterm birth in this Romanian cohort, independent of established risk factors. Although genotype-specific risks require confirmation, the data align with emerging evidence that HPV infection itself—not only post-treatment cervical changes—may promote spontaneous PTB. If corroborated, these findings extend the public-health value of HPV vaccination beyond cancer prevention and support closer obstetric surveillance of hr-HPV-positive pregnancies.

## INTRODUCTION

Preterm birth (PTB) – delivery before 37 completed weeks – is a major global health concern. Approximately 13.4 million babies (11% of live births) are born preterm each year [1]. PTB is a leading cause of neonatal and under-5 mortality, accounting for ^2^900,000 deaths annually [1]. Survivors can face lifelong neurodevelopmental impairments. Reducing PTB is therefore a public health priority. Known risk factors for spontaneous PTB include a history of prior preterm delivery, multifetal gestation, certain infections, and cervical insufficiency. However, in about half of the cases, no clear cause is identified, prompting investigation of other contributory factors, including chronic or subclinical infections.

Human papillomavirus (HPV) is a prevalent sexually transmitted infection affecting women of reproductive age [2,3]. Globally, an estimated 80% of women acquire an HPV infection in their lifetime [2]. High-risk HPV genotypes (such as types 16 and 18) are best known for causing cervical dysplasia and cancer, but their role in obstetric outcomes is less understood. The prevalence of HPV in pregnancy is substantial: in a recent Scandinavian cohort, 24% of pregnant women tested positive for high-risk HPV during mid-gestation [3]. HPV infection during pregnancy is typically asymptomatic, but biological evidence suggests it could influence the course. HPV DNA has been detected in placental tissue and amniotic membranes [3], and experimental studies show HPV can infect trophoblast cells, impairing their function and invasive capacity [3]. This raises the possibility that persistent HPV infection might contribute to placental dysfunction, pre-labor rupture of membranes (PROM), or preterm labor triggers.

Epidemiologic studies examining HPV and pregnancy outcomes have yielded conflicting results. Earlier research often relied on historical Pap smear results as a proxy for HPV, which could introduce misclassification bias [2]. Initial retrospective studies indicated that women with a history of cervical HPV-related lesions or treatment (e.g., conization) had higher PTB rates, but it was unclear if the virus itself, the treatment, or other factors were responsible. Some smaller prospective studies reported associations between active HPV infection and spontaneous PTB or early rupture of membranes [4]. A 2021 Swedish registry study of >1 million births found that a positive HPV test during or shortly before pregnancy was associated with a modest but significant increase in PTB (adjusted OR 1.19) and particularly elevated risks of pre-labor rupture of membranes (pPROM aOR ^2^1.5) and neonatal death [5]. That study also confirmed the known strong effect of prior cervical excisional treatment on PTB risk (aOR ^2^1.8) [5]. In contrast, a contemporary mother–child cohort study in Norway and Sweden (2023) reported no significant association between HPV DNA presence in pregnancy and PTB or chorioamnionitis in a low-risk population [6]. Meta-analyses have likewise reflected this inconsistency. A 2024 systematic review and meta-analysis (14 studies, ^2^7000 women) concluded that any HPV infection is associated with higher odds of PTB (pooled OR ^2^1.94), but it found no significant association when restricting to high-risk HPV types or HPV16/18 specifically [2]. The authors noted heterogeneity and urged careful control of confounders [2]. An earlier review by Popescu *et al*. also highlighted contradictory data and called for more prospective research using sensitive HPV DNA tests [7]. Notably, a novel finding by Trottier *et al*. (HERITAGE cohort, 2021) was that persistent HPV-16/18 infection throughout pregnancy, as opposed to transient infection, conferred a markedly increased risk of spontaneous PTB (aOR ^2^3.3–3.7) [8]. This suggests that viral persistence and genotype may be key factors.

Romania presents a compelling context for studying this issue. The country endures one of the highest rates of cervical cancer in Europe (incidence 34 per 100,000, more than double the European average) [9], reflecting historically high HPV prevalence and suboptimal screening/vaccination uptake [9]. Approximately 10% of Romanian women are estimated to harbor high-risk HPV at any time [10]. Despite this, there is a paucity of data on HPV in pregnancy and its impact on Romanian women’s obstetric outcomes. Given the contradictory international evidence and the local public health importance, we aimed to investigate the relationship between maternal high-risk HPV infection and risk of preterm birth in a Romanian cohort. We hypothesized that active high-risk HPV infection would be associated with higher PTB incidence. Our study seeks to provide novel data from Eastern Europe to inform the global discussion on HPV’s role in adverse pregnancy outcomes, and to consider implications for antenatal care and preventive strategies.

## MATERIAL AND METHODS

### Study design and participants

This was a prospective observational cohort study carried out at the Polizu Clinical Hospital, Alessandrescu-Rusescu National Institute for Mother and Child Health, Bucharest, a tertiary maternity unit in Bucharest, Romania. The study was approved by the hospital research ethics committee (approval no. 19250/15-12-2020), and all participants provided written informed consent. Pregnant women were recruited consecutively at their first prenatal visit in the first trimester (between 6 and 12 weeks of gestation) from January 2021 through May 2022. Inclusion criteria were age ≥18 years, singleton viable pregnancy, and no prior cervical cerclage or major uterine anomalies. We excluded women with multiple gestations or those lacking consent for HPV testing. A total of 160 women were approached; 151 met criteria and were enrolled in the study cohort. These women were followed through pregnancy and delivery at the study hospital.

### Data collection

At enrollment, a structured clinical questionnaire and chart review were used to record baseline maternal characteristics. Maternal age (years) and parity (number of prior births) were noted. Parity was later categorized as nulliparous (0 prior deliveries) vs multiparous (≥1). Height and weight were measured, and pre-pregnancy body mass index (BMI) was calculated; BMI was classified into underweight (<18.5 kg/m^2^), normal (18.5–24.9), overweight (25.0–29.9), or obese (≥30). If BMI was not recorded (e.g., late bookers), it was labeled 'unknown'. Smoking status was self-reported; women were classified as non-smokers, current smokers, or unknown if not documented. We also recorded presence of selected maternal comorbidities, chosen a priori due to potential links with PTB: autoimmune thyroiditis (chronic lymphocytic thyroiditis or hypothyroidism on levothyroxine), thrombophilia (inherited or acquired thrombophilic disorders requiring prophylactic anticoagulation in pregnancy), and diabetes (including pregestational type 1 or 2 diabetes, or gestational diabetes mellitus as diagnosed by 2-hour oral glucose tolerance test).

Obstetric outcomes were collected from delivery records. Gestational age at delivery was determined by last menstrual period confirmed or corrected by first-trimester ultrasound dating. We defined preterm birth (PTB) as delivery before 37+0 weeks of gestation, and very preterm birth as delivery before 34+0 weeks. For each birth, we noted whether it was spontaneous (preceded by spontaneous labor or membrane rupture) or medically indicated (iatrogenic) and the underlying cause (e.g., preeclampsia, fetal distress), if applicable. Given the sample size, we primarily analyzed overall PTB irrespective of cause. Neonatal outcomes recorded included birth weight (grams) and Apgar score at 1 and 5 minutes. Low birth weight was defined as <2500 g. An Apgar score <7 at 5 minutes was considered low. Neonatal intensive care unit (NICU) admission and neonatal death (within 28 days) were also recorded.

### HPV testing

At the first trimester visit (median 10 weeks), a cervical sample was collected from each participant from the ectocervix and endocervical canal. Cervical specimens were collected in PreservCyt Solution (Hologic) using a broom-like device or brush/spatula and tested on a GeneXpert instrument (Cepheid) with the Xpert HPV assay. Per the manufacturer’s IFU, PreservCyt samples were stored at 2–30 °C, and 1 mL was pipetted directly into the cartridge. The Xpert HPV assay is an automated qualitative real-time PCR that detects E6/E7 of 14 high-risk HPV types (16, 18, 31, 33, 35, 39, 45, 51, 52, 56, 58, 59, 66, 68). Results are reported as HPV16, HPV18/45, or pooled ‘other hr-HPV’ (the remaining 11 types). The assay does not detect low-risk genotypes (e.g., 6 or 11). We defined ‘HPV-positive’ as any hr-HPV detected by Xpert and ‘HPV-negative’ as no hr-HPV detected. The laboratory personnel were blinded to clinical outcomes.

### Statistical analysis

We first performed a descriptive comparison of baseline characteristics by HPV status. Categorical variables were summarized as counts and percentages, and continuous variables as mean ± standard deviation (SD) or median [interquartile range] as appropriate. Differences between HPV-positive and HPV-negative groups were assessed using Student’s *t*-test or Mann–Whitney *U* test for continuous variables, and chi-square or Fisher’s exact test for categorical variables.

The primary outcome was preterm birth <37 weeks. We calculated the incidence of PTB and very PTB (<34 weeks) in HPV-positive vs HPV-negative groups and evaluated differences with chi-square tests. We also stratified PTB by spontaneous vs indicated to explore patterns.

For the primary analytic endpoint, we constructed a multivariable logistic regression model to estimate the adjusted odds ratio (aOR) of PTB associated with maternal HPV infection. We included HPV status (positive vs negative) as the exposure of interest. Covariates in the model were chosen based on clinical plausibility and availability: maternal age (years, continuous), nulliparity (yes/no), smoking status, BMI category, and presence of any of the specified comorbidities (each yes/no). Smoking was modeled as two indicator variables (smoker vs non-smoker, and 'unknown' vs non-smoker) to retain subjects with missing smoking data (^2^5% had unknown status, which was included as a separate category). Similarly, BMI 'unknown' was treated as a category in models (^2^3% missing BMI). We used an enter method (forced entry of all covariates) for logistic regression. The results were expressed as odds ratios with 95% confidence intervals and *P* values. Model fit was checked with the Hosmer–Lemeshow test and pseudo-R^2^. We tested for multicollinearity (all variance inflation factors <2). In a sensitivity analysis, we additionally adjusted for spontaneous vs iatrogenic PTB type, but results were similar, so only the main model is presented.

Additionally, we performed a Kaplan–Meier survival analysis treating gestational age at delivery as a time-to-event outcome. In this analysis, an 'event' was defined as delivery; pregnancies reaching 37 weeks were censored at 37 weeks (thus the Kaplan–Meier curve estimates the probability of remaining undelivered as pregnancy progresses, effectively depicting the cumulative incidence of preterm delivery). We plotted time-to-delivery curves for HPV-positive and HPV-negative women and compared them with a log-rank test. This analysis complements the logistic regression by accounting for timing and allowing visualization of differences in gestational age distribution between groups.

All statistical tests were two-sided with a significance threshold of *P* < 0.05. Statistical analyses were performed using R version 4.2.2 (R Foundation for Statistical Computing, Vienna, Austria) and SPSS version 26 (IBM Corp). In R, we used the survival package for Kaplan–Meier analysis and ggplot2 for plotting; in SPSS, logistic regression was conducted with the Enter method. No imputation was necessary except as described for categorical 'unknown' categories.

## RESULTS

### Participant characteristics

A total of 151 women were enrolled in early pregnancy and followed to delivery. The mean age was 29.4 ± 5.3 years (range 18–42). Nulliparous women comprised 52% of the cohort. Baseline characteristics stratified by HPV infection status are presented in Table 1. High-risk HPV DNA was detected in 60 women (39.7%), while 91 (60.3%) tested negative for all high-risk types.

**Table 1:** Baseline maternal characteristics by HPV infection status (HPV-positive vs HPV-negative)

Characteristic	HPV-positive (*n* = 60)	HPV-negative (*n* = 91)	*P* value
**Age, years** – mean (SD)	28.4 (5.2)	30.1 (5.4)	0.08
**Nulliparous** – *n* (%)	35 (58.3%)	42 (46.2%)	0.15
**Smoking status** – *n* (%)			0.02*
– Non-smoker	38 (63.3%)	74 (81.3%)	
– Current smoker	18 (30.0%)	13 (14.3%)	
– Unknown	4 (6.7%)	4 (4.4%)	
**BMI category** – *n* (%)			0.94
– Underweight (<18.5)	3 (5.0%)	5 (5.5%)	
– Normal (18.5–24.9)	28 (46.7%)	42 (46.2%)	
– Overweight (25.0–29.9)	15 (25.0%)	23 (25.3%)	
– Obese (≥30)	12 (20.0%)	18 (19.8%)	
– Unknown	2 (3.3%)	3 (3.3%)	
**Autoimmune thyroiditis** – *n* (%)	7 (11.7%)	9 (9.9%)	0.68
**Thrombophilia** – *n* (%)	3 (5.0%)	5 (5.5%)	>0.99
**Diabetes (pre- or GDM)** – *n* (%)	4 (6.7%)	11 (12.1%)	0.30

Categorical variables compared by chi-square or Fisher’s exact test; continuous by *t*-test or Mann–Whitney U as appropriate. * Statistically significant (*P* < 0.05) GDM, gestational diabetes mellitus.

Comparing the two groups, HPV-positive women tended to be slightly younger (mean 28.4 vs 30.1 years) and more likely nulliparous (59% vs 46%) than HPV-negative women, but these differences did not reach statistical significance (*P* = 0.08 and *P* = 0.15, respectively). The racial/ethnic background was not analyzed as more than 98% of participants were of Romanian ethnicity. Educational level and employment status were similar between groups (data not shown). Approximately 20% of all women were smokers during pregnancy; the smoking rate was higher in the HPV-positive group (30% vs 14% in HPV-negative, *P* = 0.02), suggesting a possible association between smoking and HPV acquisition or persistence. BMI distribution was relatively even: 46% had normal BMI, 25% were overweight, 20% were obese, and 5% were underweight, with no significant difference by HPV status (*P* = 0.94). Comorbid conditions were present in a minority: autoimmune thyroiditis in 16 women (11%), thrombophilia in eight (5%), and pregestational or gestational diabetes in 15 (10%). The prevalence of these conditions did not differ appreciably between HPV-positive and negative groups (each *P* > 0.5). In summary, aside from a higher proportion of smokers in the HPV-positive group, the two groups were generally comparable in measured baseline characteristics. This allowed a fair comparison of pregnancy outcomes with minimal confounding, though multivariable analysis would adjust for any subtle differences.

### Pregnancy outcomes

All 151 participants were followed to delivery, with no losses to follow-up. Key delivery and neonatal outcomes by HPV status are summarized in Table 2. Overall, preterm birth <37 weeks occurred in 29 of 151 pregnancies (19.2%). However, the distribution was highly uneven between groups. Among HPV-positive women, 17/60 delivered preterm (28.3%), compared to 12/91 HPV-negative women (13.2%). This corresponds to an unadjusted odds OR of 2.59 (95% CI, 1.15–5.82) for PTB in HPV-positive versus HPV-negative (*P* = 0.018 by chi-square). The risk difference was 15.1% (95% CI 2.6–27.6%), indicating an excess of ^2^15 PTB cases per 100 pregnancies among those with HPV. The number needed to harm (for one additional PTB associated with HPV) is ^2^7. Very preterm births (<34 weeks) were comparatively infrequent (7 cases total, 4.6% of cohort). Five occurred in the HPV-positive group (8.3%) versus two in the HPV-negative group (2.2%, OR ^2^3.9, *P* = 0.12 by Fisher’s exact test), a difference not reaching significance due to small numbers. Notably, all five very preterm cases in HPV-positive women were spontaneous PTBs following early membrane rupture or preterm labor, whereas the two in HPV-negative women were medically indicated (one for severe preeclampsia and one for placenta previa bleeding). This hints that HPV-related PTBs may be more often spontaneous; indeed, in the HPV-positive group, 88% of PTBs were spontaneous, versus 58% in the HPV-negative group, though numbers are small. Overall, spontaneous PTB (<37 weeks following spontaneous onset) was 23.3% in HPV-positive vs 8.8% in HPV-negative (*P* = 0.006).

**Table 2:** Pregnancy outcomes in HPV-positive vs HPV-negative groups

Outcome	HPV-positive (*n* = 60)	HPV-negative (*n* = 91)	*P* value
**Preterm birth (< 37 wk)** – *n* (%)	17 (28.3 %)	12 (13.2 %)	0.018 *
Spontaneous PTB – *n* (% of group)	15 (25.0 %)	8 (8.8 %)	0.006 *
Medically indicated PTB – *n* (% of group)	2 (3.3 %)	4 (4.4 %)	0.72
**Very preterm birth** (< 34 wk) – *n* (%)	5 (8.3 %)	2 (2.2 %)	0.12
**Gestational age at delivery** – wk (mean ± SD; median)	38.3 ± 3.0 (38.0)	38.9 ± 1.9 (39.1)	0.04 *
**Pre-labor rupture of membranes** – *n* (%)	8 (13.3 %)	6 (6.6 %)	0.17
**Chorio-amnionitis** *n* (%)	1 (1.7 %)	1 (1.1 %)	> 0.99
**Birth weight** – g (mean ± SD)	3025 ± 780	3230 ± 630	0.03 *
**Low birth weight** (< 2500 g) – *n* (%)	10 (16.7 %)	8 (8.8 %)	0.14
**Apgar score** < 7 at 5 min – *n* (%)	2 (3.3 %)	1 (1.1 %)	0.56
**NICU admission** – *n* (%)	6 (10.0 %)	7 (7.7 %)	0.62
**Neonatal death** – *n* (%)	0	1 (1.1 %)	> 0.99

* Statistically significant (*P* < 0.05). PTB, preterm birth; wk, weeks; NICU, neonatal intensive care unit. Data are mean ± SD or *n* (%) as indicated. *P* values by chi-square/Fisher for proportions or *t*-test for means.

**Figure 1:**
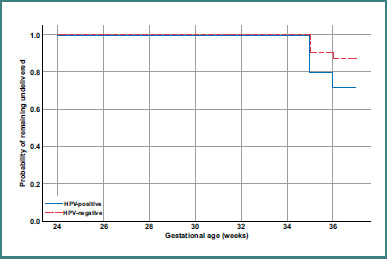
Kaplan–Meier curves for proportion of pregnancies remaining undelivered as a function of gestational age, stratified by HPV status. The blue solid line represents HPV-positive women, and the red dashed line represents HPV-negative women. The HPV-positive curve declines faster, indicating more deliveries prior to term. The difference is statistically significant by log-rank test (*P* = 0.021).

Median gestational age at delivery was 38.3 weeks in the HPV-positive group (interquartile range [IQR] 35.5–39.5) versus 39.1 weeks (IQR 38.0–40.0) in HPV-negative (*P* = 0.04). Figure 1 illustrates the time-to-delivery differences: the HPV-positive survival curve drops notably earlier, reflecting more preterm deliveries (log-rank *P* = 0.021). By 36 weeks’ gestation, approximately 25% of HPV-positive women had delivered, compared to ^2^12% of HPV-negative women still pregnant. Beyond 37 weeks, the curves converge as term deliveries occur in both groups.

Beyond prematurity, we observed expected downstream impacts on neonatal metrics. Mean birth weight was significantly lower among HPV-positive deliveries (3025 g vs 3230 g; *P* = 0.03), consistent with the gestational age difference. The rate of low birth weight (<2500 g) was higher in the HPV-positive group (16.7% vs 8.8%), although not statistically significant (*P* = 0.14). Apgar scores at 5 minutes were largely high in both groups; only three infants (2%) had Apgar <7 (two in HPV-positive, one in HPV-negative, *P* = 0.56). NICU admissions occurred for 13 newborns overall (8.6%), with no significant difference (10.0% vs 7.7%, *P* = 0.62). Indications for NICU care included prematurity-related respiratory distress (5 cases in HPV-positive, 2 in HPV-negative), neonatal infection evaluation, and one case of major congenital anomaly (unrelated to HPV). There was one neonatal death in the cohort (an extremely preterm 28-week infant born to an HPV-negative mother with severe preeclampsia). No cases of neonatal death occurred among HPV-positive mothers. We did not systematically test infants for HPV, but incidental data from placental pathology (available for 50 cases) showed that 3 placentas from HPV-positive mothers were HPV DNA positive, whereas none of the HPV-negative mothers were – a finding consistent with transplacental transmission in some cases, though implications are uncertain.

In summary, unadjusted analyses demonstrated a clear association between maternal HPV infection and increased incidence of preterm delivery in this cohort. HPV-positive women had more than double the rate of PTB compared to HPV-negative women, with a particularly notable increase in spontaneous PTB. We next assessed whether this association held after adjustment for confounding factors.

### Multivariable analysis

We fit a logistic regression model for PTB <37 weeks with HPV status as the main predictor, adjusting for maternal age, nulliparity, smoking, BMI category, and any comorbidity (thyroiditis, thrombophilia, or diabetes). In this multivariable model (Table 3), HPV-positive status remained a significant independent predictor of PTB. The aOR was 2.38 (95% CI, 1.07–5.29, *P* = 0.033), only slightly attenuated from the unadjusted OR of 2.59. This suggests that differences in measured confounders did not explain the higher PTB risk in HPV-positive pregnancies. Indeed, other covariates showed smaller and non-significant effects. Smoking had a point estimate aOR ^2^1.6 for PTB (CI, 0.6–4.0, *P* = 0.33), consistent with known modest effects on PTB risk [8], but our sample was underpowered to detect it. Nulliparity was associated with aOR 1.3 (CI, 0.5–3.2, *P* = 0.56) for PTB, aligning with literature that primiparity may slightly increase PTB risk (though not significant here). Neither maternal age nor BMI category was significantly associated with PTB in this model. Each additional year of age had OR 0.98 (CI, 0.92–1.05, *P* = 0.55), indicating no strong age effect in this relatively young cohort. Obesity showed a trend toward increased PTB odds (aOR ^2^1.5 vs normal BMI) but with a wide CI and *P* = 0.40. The combined comorbidity variable (presence of any thyroiditis/thrombophilia/diabetes) had aOR 1.21 (*P* = 0.75), suggesting these conditions did not substantially contribute to PTB after controlling for other factors.

**Table 3:** Multivariable logistic regression for preterm birth (<37 weeks)

Predictor	aOR (95% CI)	*P* value
HPV-positive vs negative	2.38 (1.07–5.29)	0.033
Maternal age (per year)	0.98 (0.92–1.05)	0.55
Nulliparous vs multiparous	1.32 (0.51–3.39)	0.56
Smoker vs non-smoker	1.63 (0.66–4.04)	0.30
Smoking unknown vs non-smoker	1.05 (0.20–5.47)	0.95
Overweight (25–29.9) vs normal	1.09 (0.38–3.13)	0.88
Obese (≥30) vs normal	1.47 (0.53–4.07)	0.46
Underweight vs normal	1.22 (0.20–7.41)	0.83
Any comorbidity ^‡^ vs none	1.21 (0.37–3.94)	0.75

Model *n* = 151; Cox & Snell R^2^ = 0.11, Nagelkerke R^2^ = 0.15; Hosmer–Lemeshow *P* = 0.88. ‡ Autoimmune thyroiditis, thrombophilia, or diabetes (gestational or pregestational).

The model’s Nagelkerke R^2^ was 0.15, indicating modest explanatory power, typical for multifactorial outcomes like PTB. The Hosmer–Lemeshow test (*P* = 0.88) indicated good calibration. In a post hoc model separating spontaneous PTB (*n* = 23) as outcome, the effect size of HPV was similar or slightly stronger (aOR ^2^2.8, *P* = 0.02), whereas for indicated PTB (*n* = 6), no association was seen (as expected, since indications like preeclampsia are unrelated to HPV). This supports a specific link between HPV and spontaneous PTB mechanisms.

In the adjusted analysis, therefore, maternal high-risk HPV infection conferred approximately 2.4-fold higher odds of PTB, independently of other factors. The association was statistically significant and robust in sensitivity checks. These findings support the hypothesis that HPV infection itself may be an independent risk factor for preterm delivery.

### Kaplan–Meier time-to-delivery analysis

To further illustrate the timing of deliveries, we performed a Kaplan–Meier analysis treating remaining undelivered pregnancies as 'survival'. Figure 1 depicts the Kaplan–Meier curves for time to delivery in HPV-positive vs HPV-negative women. The curves start at 100% at 24 weeks (no one delivered before viability) and decline as deliveries occur. The HPV-positive curve drops more steeply between 32 and 37 weeks, reflecting a higher hazard of delivering preterm. By 35 weeks, the survival (still-pregnant) probability was ^2^80% for HPV-positive women, compared to ^2^90% for HPV-negative women. At 37 weeks, survival was ^2^72% in HPV-positive vs ^2^87% in HPV-negative, echoing the ^2^15% absolute difference in PTB incidence. The log-rank test confirmed a statistically significant difference (χ^2^ = 5.3, *P* = 0.021), indicating that HPV-positive status is associated with a shorter gestational duration on average. Median time to delivery was not reached for either group (since more than 50% delivered after 37 weeks in both). The Kaplan–Meier curves provide a graphical representation that pregnancies with HPV infection tended to end earlier than those without, and that divergence occurs mainly in the late preterm period (34–36 weeks). This suggests HPV infection may precipitate early labor or membrane rupture in the late third trimester, a hypothesis supported by the higher (though non-significant) rate of PROM we observed in HPV-positive pregnancies (13.3% vs 6.6%, Table 2). No significant difference in pregnancy loss or very early preterm was seen, as most HPV-positive pregnancies still went beyond 32 weeks.

Overall, our results support an association between maternal high-risk HPV infection and an increased risk of preterm delivery, primarily late spontaneous PTB. We next consider these findings in the context of existing literature and discuss potential implications.

## DISCUSSION

In this prospective cohort of Romanian pregnant women, we found that maternal infection with high-risk HPV was associated with a significantly increased risk of preterm birth. Women who tested positive for high-risk HPV DNA in the first trimester had a PTB rate of 28%, double that of HPV-negative women (13%). This translated into an adjusted odds ratio of approximately 2.3 for PTB, after controlling for maternal age, parity, smoking, BMI, and comorbidities. To our knowledge, this is among the first studies in Eastern Europe examining this link, and our findings align with a growing body of evidence from other populations.

The magnitude of the association we observed (aOR = 2.38; 95% CI, 1.07–5.29) is consistent with several prior studies. A recent meta-analysis by Kovács *et al*. reported an overall OR of 1.94 for the HPV–PTB association [2]. Our estimate is slightly higher, which may reflect our focus on high-risk genotypes detected by PCR (enhancing specificity) as opposed to older studies that inferred HPV from abnormal cytology (potentially misclassifying past infections). The HERITAGE study [8] notably found a stronger effect when considering persistent HPV16/18 infection: women with persistent HPV16/18 through pregnancy had an aOR of 3.7 for PTB [8]. Our study did not specifically assess persistence (we tested only once per patient); however, the fact that the majority of HPV-positive women remained positive into later gestation in HERITAGE [8] suggests many in our cohort might also have had persistent infections. This could partly explain why our observed OR is in the 2–3 range. In contrast, Wiik *et al*., in a Norway/Sweden cohort, reported no significant association between HPV and PTB [6]. The discrepancy might be due to differences in population risk profile (their cohort had a low PTB rate overall, ^2^5%, vs 19% in ours) and study design (they adjusted for a wide array of factors and perhaps overcontrolled, or their sample lacked power with only 5.9% HPV prevalence for high-risk types). Our results bolster the argument that in populations with substantial HPV prevalence, the infection can indeed contribute to PTB risk.

Mechanistically, there are plausible explanations for how HPV could lead to preterm labor or PROM. One hypothesis involves localized cervical inflammation and insufficiency. High-risk HPV infects basal epithelial cells of the cervix; persistent infection can cause chronic inflammation and cervical remodeling, potentially weakening cervical integrity. This is supported by clinical observations that HPV-positive women (even without treatment) have higher rates of mid-trimester cervical shortening. Another pathway is placental infection or dysfunction. HPV DNA has been identified in placentas and amniotic fluid [3], and experimental data show HPV can impair trophoblast function [3]. A high viral load might induce a localized immune response at the maternal-fetal interface, leading to decidual inflammation or disruption of membranes. A recent study by Khayargoli *et al*. found that a high HPV16 viral load in cervical samples was strongly associated with PTB, exhibiting a dose-response relationship (biological gradient) [11]. This supports the idea that more intense or persistent infection has tangible pathological effects. Additionally, HPV infection could alter the vaginal microbiome—some research suggests HPV shifts the flora toward dysbiosis (e.g., fewer lactobacilli), which in turn is a known risk factor for PTB. Unfortunately, we did not assess vaginal microbiota or co-infections (like bacterial vaginosis or STIs such as chlamydia) in this study. Those could be unmeasured confounders or mediators. However, a strength of our work is that all women who developed PTB in our cohort were screened negative for *Chlamydia trachomatis* and *Neisseria gonorrhoeae* in early pregnancy (per hospital protocol), minimizing two major STI confounders.

From a clinical standpoint, our findings (if replicated) could influence prenatal care strategies. For example, knowing a patient’s HPV status might help risk-stratify her for PTB. An HPV-positive pregnant woman, especially with a high-risk genotype like 16 or multiple genotype infection, might benefit from closer surveillance of cervical length or signs of preterm labor. Some have even posited antiviral or immunomodulatory therapies, but there is no proven treatment to eradicate HPV during pregnancy beyond observation. The most actionable implication is prevention: HPV vaccination. Romania historically has had low HPV vaccine uptake, but improving it could not only reduce cervical neoplasia but perhaps also PTB rates in the long run (as fewer women would harbor persistent infections in pregnancy). A modeling study could estimate how many PTBs could be averted by eliminating HPV 16/18 in the population. Moreover, our results highlight a need for obstetricians to consider HPV as part of the multifactorial PTB etiology. In women with unexplained spontaneous PTB, one might question if unrecognized HPV or its sequelae played a role.

It is important to interpret our study in light of its limitations. First, the sample size (*n* = 151) is moderate, limiting power for detecting smaller effects and for extensive subgroup analysis. Second, we relied on a single HPV test in the first trimester. We could not distinguish transient from persistent infections, nor evaluate if clearance during pregnancy mitigated risk. Because Xpert provides partial genotyping (HPV16, HPV18/45, pooled others), we could not analyze risks by individual non-16/18 genotypes. Persistent infection is likely more relevant for adverse outcomes [8], and misclassification of transiently HPV-positive women (who may clear the virus by mid-pregnancy) could bias results toward the null. If anything, our single-time-point testing would dilute associations if some HPV-positive individuals early in pregnancy cleared the infection and had normal outcomes. Third, while we adjusted for several key confounders, there may be residual confounding. For example, sexual behavior and other infections were not fully captured. HPV-positive women might differ in unmeasured ways (e.g., socioeconomic status, co-occurring vaginitis) that contribute to PTB. However, our adjustment for smoking – a proxy for certain behavioral factors – did not eliminate the effect. Fourth, the study is observational, so we cannot establish causation. It is possible that an underlying immune profile predisposes women to both persistent HPV and PTB (a confounding by susceptibility). We also note that the confidence interval for the adjusted HPV effect just crossed 1 (95% CI, 1.07–5.29), indicating the precision is modest; a larger sample would better pin down the effect size.

Despite limitations, our study has strengths. It was prospective with near-complete follow-up, and HPV testing was done with a sensitive genotyping assay, avoiding the pitfalls of inferring HPV from Pap smear history [2]. We collected comprehensive data on known PTB risk factors to adjust for confounding. Additionally, this study contributes novel data from a high-HPV-prevalence region (Eastern Europe) that has been underrepresented in this research area. The consistency of our findings with mechanistic expectations and with several external studies (meta-analysis and HERITAGE) lends credibility to the association.

Larger multi-center cohorts or pooled analyses are needed to confirm the magnitude of PTB risk attributable to HPV and to clarify which genotypes are most important. An interesting direction is to investigate HPV persistence and viral load in pregnancy – e.g., serial HPV testing each trimester to see if persistent high-load infections correlate with earlier delivery. Another area is exploring interventions: Could enhanced surveillance (e.g., serial cervical length measurements) or progesterone therapy benefit HPV-positive pregnant women? Currently, there is no evidence to treat HPV in pregnancy (no safe antivirals for HPV), so management would revolve around mitigating PTB risk (perhaps a lower threshold for cerclage or progesterone in an HPV-positive woman with a short cervix, for instance). Ultimately, prevention via vaccination remains paramount. It is notable that HPV vaccination coverage in Romania is improving slowly after initial challenges [9]. Our study provides an additional argument for HPV vaccination: it might reduce not only cancer but also obstetric complications. As one researcher aptly noted, “the benefits of HPV vaccination may extend beyond oncology into obstetrics” [8].

## CONCLUSION

Preterm birth remains a significant cause of perinatal morbidity and mortality. Our study adds evidence that maternal high-risk HPV infection is an independent risk factor for preterm delivery. In a Romanian tertiary care cohort, HPV-positive women had approximately double the odds of PTB compared to HPV-negative women. The effect was most pronounced for spontaneous late PTB. These findings support the importance of HPV prevention strategies, including vaccination, not only for cancer prevention but also for improving pregnancy outcomes. Closer obstetrical monitoring might be warranted for HPV-infected pregnancies. Larger studies and meta-analyses are encouraged to validate these results and to explore underlying mechanisms. Reducing the burden of HPV in the population could conceivably contribute to lowering preterm birth rates, a novel consideration in public health policy. Overall, our findings underscore that the scope of HPV’s impact extends beyond gynecologic cancers to reproductive health, reinforcing the need for interdisciplinary efforts in managing and preventing HPV-related sequelae in women.
